# Genetically predicted ankylosing spondylitis increases the risk of heart failure: a Mendelian randomization study

**DOI:** 10.3389/fcvm.2025.1415263

**Published:** 2025-07-22

**Authors:** Chennan Liu, Laicheng Wang, Mei Lin, Jing You, Xuan Zheng, Ailing Wang, Yunchai Lin, Feng Peng, Dan Hu

**Affiliations:** ^1^ Department of Cardiology, The First Affiliated Hospital of Fujian Medical University, Fuzhou, China; ^2^ Department of Cardiology, National Regional Medical Center, Binhai Campus of the First Affiliated Hospital, Fujian Medical University, Fuzhou, China; ^3^ Department of Cardiology, Fujian Medical University Union Hospital, Fuzhou, China; ^4^ Department of Pathology, Clinical Oncology School of Fujian Medical University, Fujian Cancer Hospital, Fuzhou, Fujian, China

**Keywords:** ankylosing spondylitis, Mendelian randomization, cardiovascular disease, heart failure, the causal link

## Abstract

**Background:**

Observational studies have indicated a potential association between ankylosing spondylitis and elevated cardiovascular disease (CVD) risk. However, it remains uncertain whether this association reflects a causal association. Therefore, we conducted this Mendelian randomization to investigate the causal relationship between ankylosing spondylitis and CVD.

**Methods:**

We performed a two-sample Mendelian randomization analysis to establish a causal relationship between ankylosing spondylitis and the risk of six prevalent CVDs: hypertension, heart failure, atrial fibrillation, coronary heart disease, myocardial infarction, and peripheral artery disease. Furthermore, multivariate Mendelian randomization was employed to determine the influence of confounding factors.

**Results:**

Genetically predicted ankylosing spondylitis increased the risk of heart failure according to the Mendelian randomization analysis (OR = 1.02; 95% CI = 1.00–1.04; *p* = 0.030). Multivariate analysis results indicated that the causal relationship between ankylosing spondylitis and heart failure risk remained significant after adjusting for hypertension, diabetes, smoking, body mass index, and pulse wave reflection index. The associations diminished and lost significance after adjusting for pulse wave peak-to-peak time and pulse wave arterial stiffness index.

**Conclusion:**

Genetically predicted ankylosing spondylitis increased the risk of heart failure, and the causality may partly be mediated by increased arterial stiffness.

## Introduction

Ankylosing spondylitis is an emerging interdisciplinary subject, with cardio-rheumatology aiming to explore the relationship between rheumatic diseases and cardiovascular diseases (CVDs) (1). It is generally recognized that patients with inflammatory rheumatic diseases have a higher incidence of cardiovascular complications (2). Meta-analyses have indicated that people with rheumatoid arthritis have a 48% increased cardiovascular disease (CVD) risk (3). In addition, patients with ankylosing spondylitis and psoriatic arthritis may be associated with increased CVD risk (4, 5). The role of chronic systemic inflammation and autoimmunity in this process is significant, although the precise pathophysiological mechanisms remain incompletely elucidated. Furthermore, traditional cardiovascular risk factors, including smoking, obesity, and others, along with medications, may also contribute to this phenomenon. Therefore, clarifying the causal relationship between rheumatic diseases and CVDs and exploring their mediating factors stands as highly significant and has great potential to improve the prognosis for patients with rheumatic disease.

Ankylosing spondylitis is an inflammatory arthritis that is distinguished by inflammation and excessive bone growth in the axial skeleton, resulting in limited mobility and potential lifelong disability (6). Furthermore, ankylosing spondylitis frequently presents with extra-articular manifestations, including anterior uveitis, inflammatory bowel disease, aortic valve insufficiency, and cardiac conduction disorder (7). The involvement of cardiovascular complications significantly influences the progression of ankylosing spondylitis. Cardiac complications, such as aortitis, valvular heart diseases, and cardiac conduction abnormalities, are observed in 10%–30% of individuals with ankylosing spondylitis (8). Ankylosing spondylitis patients face a higher risk of mortality due to cardiovascular diseases (9). However, it is important to note that the vast majority of the existing research is derived from observational studies, which are susceptible to confounding factors and reverse causality (10).

Mendelian randomization (MR) analysis is a novel epidemiological tool to avoid potential unmeasured confounders and to confirm whether a trait is causally associated with an outcome by incorporating single-nucleotide polymorphisms (SNPs) as a diagnostic tool (11, 12). Therefore, Mendelian randomization analysis has been widely used to investigate the causality of disease. Nevertheless, few Mendelian randomization studies have focused on the association between ankylosing spondylitis and cardiovascular outcomes. Therefore, we performed univariate analysis to clarify the causality between ankylosing spondylitis and six common CVDs, including hypertension, heart failure, atrial fibrillation, coronary heart disease, myocardial infarction, and peripheral artery disease. For the positive outcome in the univariate analysis, we further performed multivariate analysis to assess the influence of confounders or mediators.

## Methods

### Study design

**Figure 1:**
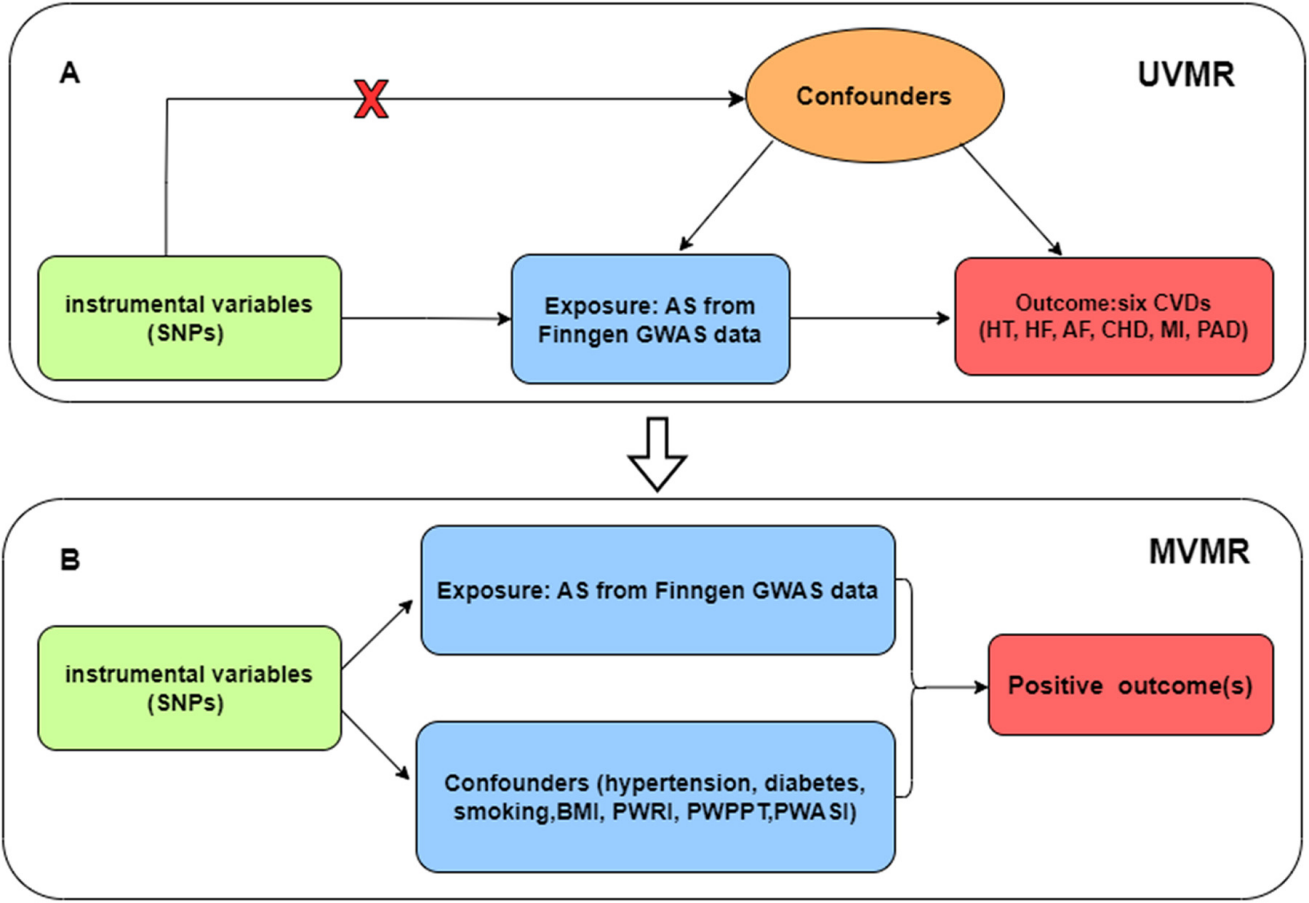
**(A)** Univariate Mendelian randomization; **(B)** Multivariate Mendelian randomization. Three main assumptions of Mendelian randomization analysis need to be followed: (1) Genetic instrumental variables (IVs) were strongly correlated with exposure (AS); (2) IVs were independent of any confounding factors; (3) IVs had effects on the outcomes (CVD) only via exposure (AS). SNP, single-nucleotide polymorphism; UVMR, univariate Mendelian randomization; MVMR, multivariate Mendelian randomization; AS, ankylosing spondylitis; HT, hypertension; HF, heart failure; AF, atrial fibrillation; CHD, coronary heart disease; MI, myocardial infarction; PAD, peripheral artery disease; BMI, body mass index; PWRI, pulse wave reflection index; PWPPT, pulse wave peak -to-peak time; PWASI, pulse wave arterial stiffness index.

We performed univariate analysis to assess the potential causal association between ankylosing spondylitis and CVDs (including hypertension, heart failure, atrial fibrillation, coronary heart disease, myocardial infarction, and peripheral artery disease). For the positive outcomes, multivariate analysis was used to search for confounders or mediators. The flowchart of our study design is presented in Figure 1.

### Selection of instrumental variables

Firstly, we extracted the genetic instruments of exposure at a strict threshold (*p* < 5 × 10^−8^). To remove linkage disequilibrium (LD), we chose the clumping window of *r*^2^ = 0.01 and a genetic distance of 10,000 kb. Secondly, we harmonized the filtered data to exclude palindromic SNPs. We examined the association between selected SNPs and potential confounders by PhenoScanner (13). Finally, to ensure that the SNPs selected have sufficient statistical strength, we used the following formula: *F* = R2 × (*N*–*k*–1)/[(1–R2) × *k*], where R2 = 2 × *β*2 × (1–EAF) × EAF to calculate the *F* statistics for each SNP (*N* = sample size, *k* = the number of SNPs). Instrumental variables (IVs) with low statistical power (*F* < 10) were removed from Mendelian randomization analysis (14). Detailed information on selected SNPs is presented in Supplementary Table 1.

### Statistical analysis

The statistical analysis was carried out using R software (version 4.3.0) and TwoSampleMR (version 0.5.7).

The inverse variance weighted (IVW) method was used to perform the Mendelian randomization analysis since it is conventionally the most reliable when the total IVs are available (10). The random-effects IVW technique was applied to obtain a more accurate estimation when heterogeneity is present (12). As a part of the reliability assessment, MR-Egger regression (15) and weighted median (16) were also conducted. To confirm that the results were valid and robust, we applied multiple methods for sensitivity analysis. A Cochrane's *Q* test was used to evaluate heterogeneity, and if heterogeneity was detected (*p* < 0.05), we used the random-effects IVW model to ensure a conservative outcome (17). Our MR-Egger regression was used to assess whether there was pleiotropy on the horizontal scale, with the intercept term showing how pleiotropy was distributed across the IVs (15). Outliers in IVW linear regression were identified using MR pleiotropy residual sum and outlier (MR-PRESSO), which corrects estimates by removing outliers when they are present (18). Furthermore, the results of Mendelian randomization analysis were examined for robustness and reliability using the leave-one-out method.

As the univariate analysis we conducted may be confounded or mediated by common cardiovascular risks, we performed multivariate analysis, adjusting for hypertension, diabetes, body mass index, smoking, and arteriosclerosis-related characteristics (pulse wave reflection index, pulse wave peak-to-peak time, pulse wave arterial stiffness index). Based on the same criteria, we obtained instrumented variables for possible risk factors and conducted multivariate analysis with the exposure data of ankylosing spondylitis to assess the impact on outcomes.

## Results

### Data source

Genome-wide association study (GWAS) data on ankylosing spondylitis were derived from the FinnGen database, which contains 166,144 individuals of European ancestry (1,462 cases and 164,682 controls). All ankylosing spondylitis cases followed the definition of M13 code in the International Classification of Diseases (ICD) 10. We derived the data on heart failure from the Heart Failure Molecular Therapeutic Target Epidemiology (HERMES) Consortium, containing 977,323 European individuals (47,309 cases and 930,014 controls) (19). The GWAS summary data on hypertension were from the UK Biobank, including 91,033 cases and 245,650 controls. The data source of atrial fibrillation was accessed from a summary GWAS meta-analysis by Roselli et al (20), incorporating 537,409 individuals of European ancestry (55,114 cases and 482,295 controls). We derived the summary data on coronary heart disease from the Coronary Artery Disease Genetics Consortium, which consisted of 60,801 patients and 123,504 controls (21). The genetic data on myocardial infarction were obtained from a large-scale GWAS analysis, including 395,795 individuals of European ancestry (14,825 cases and 380,970 controls) (22). The data source of peripheral artery disease was accessed from the UK Biobank, containing 1,230 cases and 359,964 controls. All summary statistics for ankylosing spondylitis and six CVDs were accessed from the IEU OpenGWAS database. Table 1 shows the details of the datasets used in this study.

**Table 1:** Data source.

Phenotypes	Data source	Phenotypic code	Cases/controls	Population
Exposure				
AS	FinnGen	finn-b-M13_ANKYLOSPON	1,462/164,682	European
Outcome				
HT	UK Biobank	ukb-a-437	91,033/245,650	European
HF	Shah S	ebi-a-GCST009541	47,309/930,014	European
AF	Roselli C	ebi-a-GCST006061	55,114/482,295	European
CHD	CARDIoGRAMplusC4D	ieu-a-7	60,801/123,504	Mixed (77% European)
MI	Hartiala JA	ebi-a-GCST011365	14,825/ 380,970	European
PAD	UK Biobank	ukb-d-I9_PAD	1,230/359,964	European
Confounder				
HT	MRC-IEU	ukb-b-14177	124,227/337,653	European
Diabetes	MRC-IEU	ukb-b-10753	22,340/439,238	European
Smoking	MRC-IEU	ukb-b-20261	280,508/180,558	European
BMI	MRC-IEU	ukb-b-19953	461,460	European
PWRI	MRC-IEU	ukb-b-11598	151,546	European
PWPPT	MRC-IEU	ukb-b-8778	151,466	European
PWASI	MRC-IEU	ukb-b-11971	151,053	European

AS, ankylosing spondylitis; HT, hypertension; HF, heart failure; AF, atrial fibrillation; CHD, coronary heart disease; MI, myocardial infarction; PAD, peripheral artery disease; BMI, body mass index; PWRI, pulse wave reflection index; PWPPT, pulse wave peak-to-peak time; PWASI, pulse wave arterial stiffness index.

### Causal estimates of genetic susceptibility to ankylosing spondylitis and CVD risk

**Figure 2:**
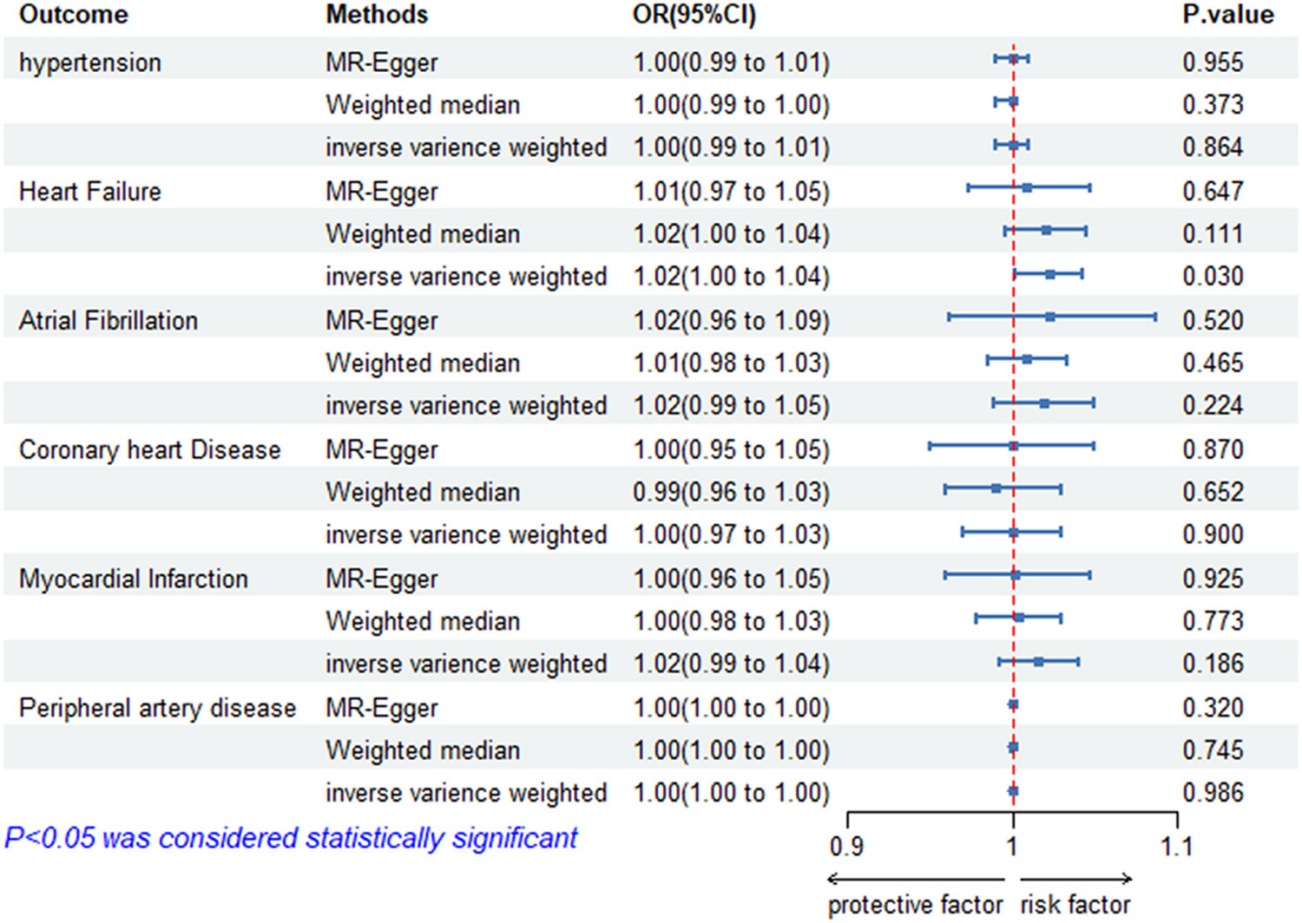
Risk of CVDs for individuals with genetically predicted ankylosing spondylitis. OR, odds ratio; CI, confidence interval; HT, hypertension; HF, heart failure; AF, atrial fibrillation; CHD, coronary heart disease; MI, myocardial infarction; PAD, peripheral artery disease; AS, ankylosing spondylitis.

According to the IVW model, there is evidence to suggest that genetically predicted ankylosing spondylitis has a significant causal impact on heart failure. Patients with ankylosing spondylitis have an increased risk of heart failure compared with those without ankylosing spondylitis (OR = 1.02; 95% CI = 1.00–1.04; *p* = 0.030). The effect estimates obtained from the weighted median and MR-Egger models also generally align with the findings of the IVW model. Nevertheless, we did not find a significant causal relationship between ankylosing spondylitis and other cardiovascular diseases, such as hypertension (OR = 1.00; 95% Cl = 0.99–1.01; *p* = 0.864), atrial fibrillation (OR = 1.02, 95% Cl = 0.99–1.05; *p* = 0.224), and myocardial infarction (OR = 1.02; 95% Cl = 0.99–1.04; *p* = 0.186). All the univariate results between ankylosing spondylitis and six CVDs are shown in Figure 2.

### Sensitivity analysis

The sensitivity analysis results are presented in Table 2. Based on IVW Cochran's *Q* test, none of the causal effects analyses showed heterogeneity among the used SNPs, except for ankylosing spondylitis—hypertension (Cochrane's *Q* = 11.329, *p* = 0.023) and ankylosing spondylitis—atrial fibrillation (Cochrane's *Q* = 9.57, *p* = 0.048). In Egger regression analysis, there was no horizontal pleiotropy detected since all Egger intercepts exceeded 0.05. According to the MR-PRESSO test, there were no outliers in the causal analysis, indicating that the estimated causal relationships in this study are reliable. In addition, the funnel plots, scatter plots, and leave-one-out analysis for each association pair can be found in the Supplementary Material.

**Table 2:** Sensitivity analysis.

MR analysis	Heterogeneity test			Pleiotropy	se	pval	MR-PRESSO
	Q_pval	Q_df	Q	Egger_intercept			Global test *p*-value
AS-HT	0.023	4	11.329	7.65 × 10^−5^	0.003	0.98	0.086
AS-HF	0.95	5	1.139	0.0086	0.01	0.455	0.9593
AS-AF	0.048	4	9.57	−0.0026	0.017	0.89	0.1098
AS-CHD	0.6287	4	2.589	−0.004	0.013	0.78	0.6282
AS-MI	0.268	4	5.193	0.00897	0.0123	0.519	0.3441
AS-PAD	0.242	4	5.47	0.00027	0.0001	0.24	0.2754

MR, Mendelian randomization; AS, ankylosing spondylitis; HT, hypertension; HF, heart failure; AF, atrial fibrillation; CHD, coronary heart disease; MI, myocardial infarction; PAD, peripheral artery disease.

### Multivariate Mendelian randomization

We found the causal relationship between ankylosing spondylitis and heart failure in the univariate analysis. Given that potential confounders may interfere with the causal inference, we conducted a multivariate analysis to explore the direct effect of ankylosing spondylitis on heart failure risk (Table 3).

**Table 3:** Results from multivariate Mendelian randomization.

Exposure	Model	Outcome	OR (95% Cl)	*p*
AS	MVMR (adjusted for hypertension)	HF	1.015 (1.005–1.024)	0.00113
	MVMR (adjusted for diabetes)		1.017 (1.002–1.032)	0.028
	MVMR (adjusted for smoking)		1.016 (1.008–1.023)	7.54 × 10^−5^
	MVMR (adjusted for BMI)		1.014 (1.006–1.021)	4.18 × 10^−4^
	MVMR (adjusted for PWRI		1.015 (1.003–1.026)	0.013
	MVMR (adjusted for PWPPT)		1.016 (0.999–1.032)	0.052
	MVMR (adjusted for PWASI		1.017 (0.998–1.035)	0.067

AS, ankylosing spondylitis; HF, heart failure; BMI, body mass index; PWRI, pulse wave reflection index; PWPPT, pulse wave peak-to-peak time; PWASI, pulse wave arterial stiffness index; MVMR, multivariate Mendelian randomization.

In the multivariate analysis, the results of our study indicate that the causal association between ankylosing spondylitis and the risk of heart failure remained statistically significant even after controlling for hypertension (OR = 1.015; 95% CI = 1.006–1.024; *p* = 1.13 × 10^−3^), diabetes (OR = 1.017; 95% CI = 1.002–1.032; *p* = 0.028), smoking (OR = 1.016; 95% CI = 1.008–1.024; *p* = 7.54 × 10^−5^), body mass index (OR = 1.014; 95% CI = 1.006–1.022; *p* = 4.18 × 10^−4^), and pulse wave reflection index (OR = 1.015; 95% CI = 1.003–1.026; *p* = 0.013). However, after adjusting for pulse wave peak-to-peak time (OR = 1.016; 95% CI = 0.999–1.032; *p* = 0.052) and pulse wave arterial stiffness index (OR = 1.017; 95% CI = 0.998–1.036; *p* = 0.067), the causal association between ankylosing spondylitis and heart failure became less significant.

## Discussion

To the best of our knowledge, this study represents the investigation into the causal association between ankylosing spondylitis and multiple CVDs utilizing Mendelian randomization analysis. Our findings suggest that ankylosing spondylitis might serve as a risk factor for heart failure, and no analogous significant relationship was observed with other cardiovascular outcomes. Moreover, upon adjusting for arterial stiffness-related characteristics, the previously observed significant causal impact of ankylosing spondylitis on heart failure became non-significant.

Previous studies have documented the correlation between ankylosing spondylitis and susceptibility to CVDs. An observational study including 14,129 patients newly diagnosed with ankylosing spondylitis has posited that ankylosing spondylitis is an independent risk factor for atrial fibrillation (23). Additionally, patients with ankylosing spondylitis had more enhanced monocyte–endothelial interactions, which can accelerate atherogenesis (24). Nevertheless, it is important to acknowledge that these observational studies are prone to confounding variables and reverse causation, and our Mendelian randomization analysis did not yield significant causal associations. Regarding heart failure, prior observational studies have reported the association between ankylosing spondylitis and heart failure. A cohort study including 12,988 ankylosing spondylitis cases suggested that patients with ankylosing spondylitis are at a heightened risk of developing congestive heart failure, consistent with our findings (25). Nevertheless, the precise mechanism underlying the increased occurrence of heart failure in ankylosing spondylitis patients remains unclear.

Studies have demonstrated a higher incidence of aortic valve disease and heart failure among ankylosing spondylitis patients when compared with the general population (26). Damage to the aortic valve is a frequently observed complication of ankylosing spondylitis (8). This damage often manifests as valve thickening, insufficiency, and regurgitation (27). The impaired function of the valve has a significant impact on the hemodynamics, potentially leading to an increased burden on the heart and the development of heart failure. Additionally, aortitis, primarily affecting the aortic ring and ascending aorta, is a common occurrence in ankylosing spondylitis patients (28). Furthermore, individuals with ankylosing spondylitis have an elevated prevalence of heart conduction disorders, particularly atrioventricular block, with the conduction abnormality potentially being linked to the presence of human leukocyte antigen B27 (27). Conduction disorders affect the rhythm and ejection of the heart, increasing the risk of heart failure.

In our multivariate analysis, it was found that the causal effect of ankylosing spondylitis on heart failure lost significance upon adjustment for pulse wave peak-to-peak time and pulse wave arterial stiffness index, which serve as measurements of arterial stiffness. This finding suggested that increased arterial stiffness may play a partial mediating role in the process from ankylosing spondylitis to heart failure. Prior systemic review had demonstrated that an elevation in arterial stiffness among individuals with chronic inflammatory and autoimmune diseases, including ankylosing spondylitis (29). Furthermore, a case–control study indicated a strong association between disease activity and arterial stiffness levels in ankylosing spondylitis patients (30). Increased arterial stiffness in chronic inflammatory rheumatic diseases is a leading cause of diastolic dysfunction, which serves as one of the primary mechanisms for the development of heart failure (29). Consequently, early screening and intervention measures for atherosclerosis can mitigate the incidence of cardiovascular events in ankylosing spondylitis patients, including heart failure.

A previous Mendelian study by Zhong et al. (32) pointed out that there is no causal relationship between ankylosing spondylitis and cardiovascular diseases. They used the genetic data of the GWAS study on ankylosing spondylitis published by Crotes et al. (31) in 2013, which included a total of 22,647 European participants (9,069 cases and 13,578 controls) (31). In our research, the genetic data for ankylosing spondylitis were derived from the 2021 FinnGen dataset, which contains 166,144 individuals of European ancestry (1,462 cases and 164,682 controls). Compared with the study by Zhong et al. (32), our exposure data were obtained from a different cohort, and the data were more recent with a larger sample size. We found a significant genetic association between ankylosing spondylitis and heart failure. We applied multivariate analysis to further demonstrate the reliability of our result for the first time, which had not been implemented in previous similar Mendelian randomization studies.

Inevitably, certain limitations should be acknowledged. For the outcomes, we did not find data on specific subtypes, such as the types of heart failure classified by ejection fraction. The study population consisted mostly of individuals with European ancestry, and we do not know if we can get similar results in other populations. Lastly, the relatively low values of ORs in this study indicate that the results should be interpreted with caution.

## Conclusion

Our study revealed that genetically predicted ankylosing spondylitis increases the risk of heart failure. Nevertheless, ankylosing spondylitis and other cardiovascular outcomes did not show a causal relationship in our investigation. The connection between ankylosing spondylitis and heart failure may be mediated by increased arterial stiffness, implying that early screening and intervention for atherosclerosis might mitigate the occurrence of heart failure and enhance the quality of life of individuals with ankylosing spondylitis.

## Data Availability

The original contributions presented in the study are included in the article/Supplementary Material, further inquiries can be directed to the corresponding authors.
