# Supplementary material for: Genetically predicted ankylosing spondylitis increases the risk of heart failure: a Mendelian randomization study
Source: Front Cardiovasc Med. 2025 Jul 22;12:1415263. doi: 10.3389/fcvm.2025.1415263 (PMC12321883; doi:10.3389/fcvm.2025.1415263)
Supplement: Supplementary file 1 [file Datasheet1.docx]

***Supplementary Material***

Supplementary table1: detailed information of selected SNPs

MR analysis of Ankylosing spondylitis and Hypertension

| SNP | A1 | A2 | EAF | Ankylosing spondylitis | | | Hypertension | | | R^2^ | F |
| --- | --- | --- | --- | --- | --- | --- | --- | --- | --- | --- | --- |
|  |  |  |  | Beta | SE | P-val | Beta | SE | P-val |  |  |
| rs10807943 | C | T | 0.94 | -0.56 | 0.081 | 4.10E-12 | -7.00E-04 | 1.73E-03 | 0.68 | 0.038 | 47.22 |
| rs13033284 | C | T | 0.63 | -0.22 | 0.039 | 9.67E-09 | 4.58E-04 | 1.11E-03 | 0.68 | 0.023 | 17.45 |
| rs34982906 | C | T | 0.052 | 0.82 | 0.092 | 3.82E-19 | 8.98E-03 | 2.96E-03 | 2.45E-03 | 0.067 | 151.69 |
| rs76644067 | A | G | 0.047 | 0.73 | 0.095 | 9.44E-15 | -5.5E-04 | 2.78E-03 | 0.84 | 0.049 | 78.48 |
| rs79693223 | T | C | 0.045 | 1.30 | 0.11 | 3.36E-34 | -3.80E-03 | 2.78E-03 | 0.17 | 0.14 | 706.88 |

MR analysis of Ankylosing spondylitis and Heart failure

| SNP | A1 | A2 | EAF | Ankylosing spondylitis | | | Heart failure | | | R^2^ | F |
| --- | --- | --- | --- | --- | --- | --- | --- | --- | --- | --- | --- |
|  |  |  |  | Beta | SE | P-val | Beta | SE | P-val |  |  |
| rs10807943 | C | T | 0.94 | -0.56 | 0.081 | 4.10E-12 | -0.015 | 0.016 | 0.35 | 0.038 | 39.35 |
| rs13033284 | C | T | 0.63 | -0.22 | 0.039 | 9.67E-09 | -0.012 | 8.1E-03 | 0.13 | 0.0229 | 14.54 |
| rs34982906 | C | T | 0.052 | 0.82 | 0.092 | 3.82E-19 | 0.022 | 0.024 | 0.36 | 0.067 | 126.40 |
| rs76644067 | A | G | 0.047 | 0.73 | 0.095 | 9.44E-15 | 0.015 | 0.021 | 0.46 | 0.049 | 65.40 |
| rs79693223 | T | C | 0.045 | 1.30 | 0.11 | 3.36E-34 | 0.023 | 0.022 | 0.28 | 0.14 | 589.06 |
| rs9264277 | C | T | 0.73 | 0.52 | 0.045 | 3.73E-31 | 5.30E-03 | 0.014 | 0.70 | 0.11 | 318.17 |

MR analysis of Ankylosing spondylitis and Atrial fibrillation

| SNP | A1 | A2 | EAF | Ankylosing spondylitis | | | Atrial fibrillation | | | R^2^ | F |
| --- | --- | --- | --- | --- | --- | --- | --- | --- | --- | --- | --- |
|  |  |  |  | Beta | SE | P-val | Beta | SE | P-val |  |  |
| rs10807943 | C | T | 0.94 | -0.56 | 0.081 | 4.10E-12 | 4.00E-04 | 0.012 | 0.98 | 0.038 | 47.22 |
| rs13033284 | C | T | 0.63 | -0.22 | 0.039 | 9.67E-09 | -3.20E-03 | 7.30E-03 | 0.66 | 0.023 | 17.45 |
| rs34982906 | C | T | 0.052 | 0.82 | 0.092 | 3.82E-19 | 0.081 | 0.023 | 4.25E-04 | 0.067 | 151.69 |
| rs76644067 | A | G | 0.047 | 0.73 | 0.095 | 9.44E-15 | 2.80E-03 | 0.018 | 0.88 | 0.049 | 78.48 |
| rs79693223 | T | C | 0.045 | 1.30 | 0.11 | 3.36E-34 | 0.015 | 0.021 | 0.48 | 0.14 | 706.88 |

MR analysis of Ankylosing spondylitis and Coronary heart disease

| SNP | A1 | A2 | EAF | Ankylosing spondylitis | | | Coronary heart disease | | | R^2^ | F |
| --- | --- | --- | --- | --- | --- | --- | --- | --- | --- | --- | --- |
|  |  |  |  | Beta | SE | P-val | Beta | SE | P-val |  |  |
| rs10807943 | C | T | 0.94 | -0.56 | 0.081 | 4.10E-12 | 0.017 | 0.017 | 0.31 | 0.038 | 47.22 |
| rs13033284 | C | T | 0.63 | -0.22 | 0.039 | 9.67E-09 | 2.40E-03 | 9.8E-03 | 0.80 | 0.023 | 17.45 |
| rs34982906 | C | T | 0.052 | 0.82 | 0.092 | 3.82E-19 | 8.60E-03 | 0.033 | 0.79 | 0.067 | 151.69 |
| rs76644067 | A | G | 0.047 | 0.73 | 0.095 | 9.44E-15 | 0.025 | 0.022 | 0.25 | 0.049 | 78.48 |
| rs79693223 | T | C | 0.045 | 1.30 | 0.11 | 3.36E-34 | -0.012 | 0.033 | 0.72 | 0.14 | 706.88 |

MR analysis of Ankylosing spondylitis and Myocardial infarction

| SNP | A1 | A2 | EAF | Ankylosing spondylitis | | | Myocardial infarction | | | R^2^ | F |
| --- | --- | --- | --- | --- | --- | --- | --- | --- | --- | --- | --- |
|  |  |  |  | Beta | SE | P-val | Beta | SE | P-val |  |  |
| rs10807943 | C | T | 0.94 | -0.56 | 0.081 | 4.10E-12 | -2.08E-03 | 0.013 | 0.87 | 0.038 | 47.22 |
| rs13033284 | C | T | 0.63 | -0.22 | 0.039 | 9.67E-09 | -0.010 | 7.63E-03 | 0.19 | 0.023 | 17.45 |
| rs34982906 | C | T | 0.052 | 0.82 | 0.092 | 3.82E-19 | 0.056 | 0.023 | 0.017 | 0.067 | 151.69 |
| rs76644067 | A | G | 0.047 | 0.73 | 0.095 | 9.44E-15 | 4.08E-03 | 0.018 | 0.82 | 0.049 | 78.48 |
| rs79693223 | T | C | 0.045 | 1.30 | 0.11 | 3.36E-34 | 0.0022 | 0.022 | 0.92 | 0.14 | 706.88 |

MR analysis of Ankylosing spondylitis and Peripheral artery disease

| SNP | A1 | A2 | EAF | Ankylosing spondylitis | | | Peripheral artery disease | | | R^2^ | F |
| --- | --- | --- | --- | --- | --- | --- | --- | --- | --- | --- | --- |
|  |  |  |  | Beta | SE | P-val | Beta | SE | P-val |  |  |
| rs10807943 | C | T | 0.94 | -0.56 | 0.081 | 4.10E-12 | 2.37E-05 | 2.10E-04 | 0.91 | 0.038 | 47.22 |
| rs13033284 | C | T | 0.63 | -0.22 | 0.039 | 9.67E-09 | -1.70E-04 | 1.40E-04 | 0.24 | 0.023 | 17.45 |
| rs34982906 | C | T | 0.052 | 0.82 | 0.092 | 3.82E-19 | 5.41E-04 | 3.70E-04 | 0.15 | 0.067 | 151.69 |
| rs76644067 | A | G | 0.047 | 0.73 | 0.095 | 9.44E-15 | 8.34E-05 | 3.50E-04 | 0.81 | 0.049 | 78.48 |
| rs79693223 | T | C | 0.045 | 1.30 | 0.11 | 3.36E-34 | -4.90E-04 | 3.50E-04 | 0.17 | 0.14 | 706.88 |

A B


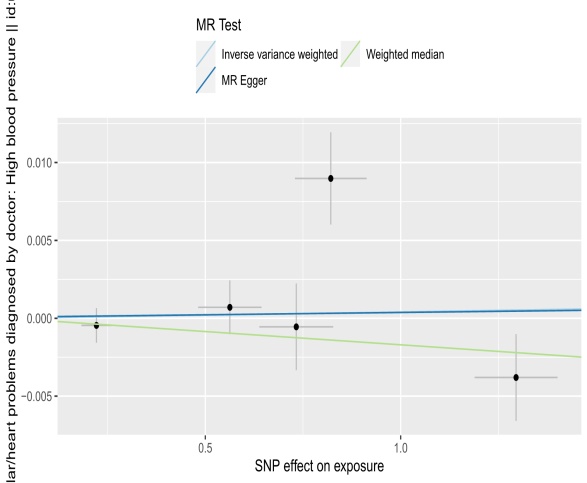

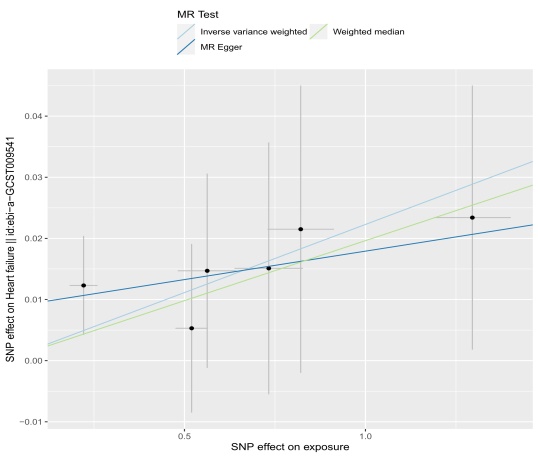


C D


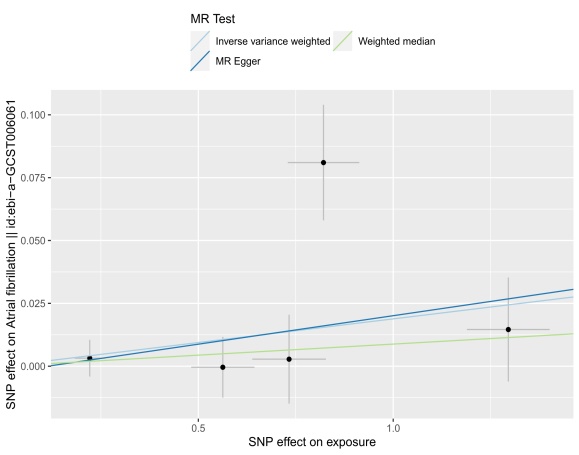

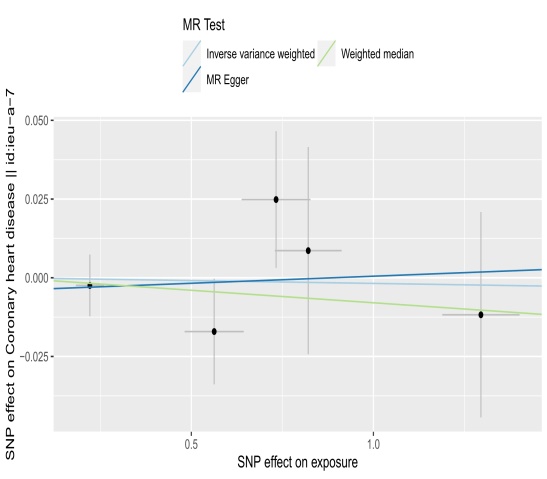


E F


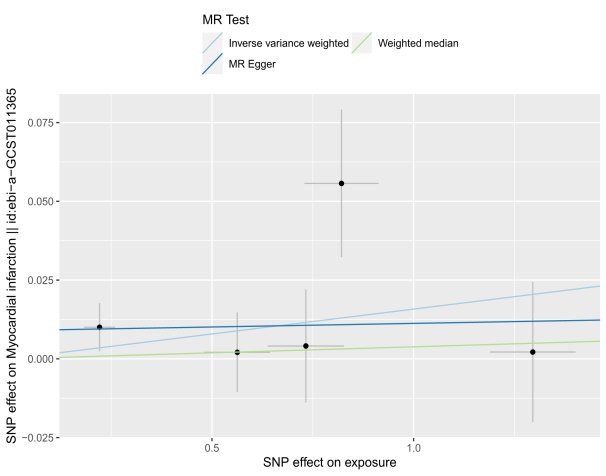

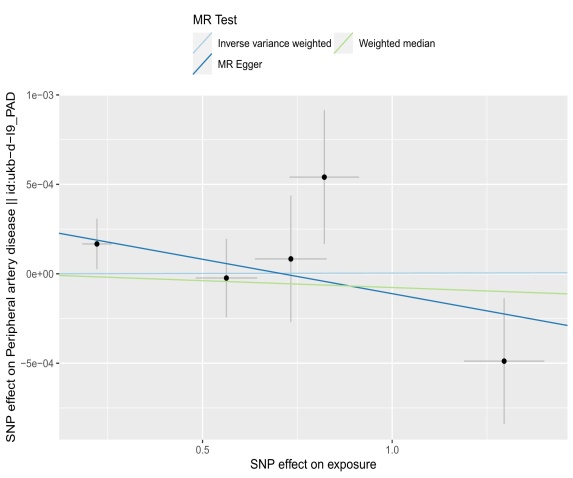


Supplementary Figure 1. Scatter plot using all IVs. (A) AS and hypertension; (B) AS and heart failure; (C) AS and atrial fibrillation; (D) AS and coronary heart disease; (E) AS and myocardial infarction. (F) AS and peripheral artery disease.

A B


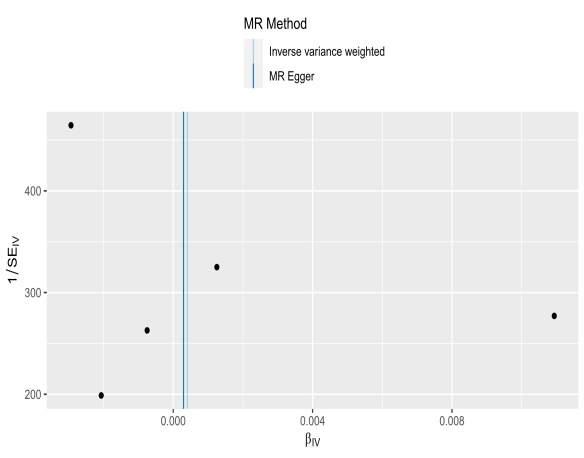

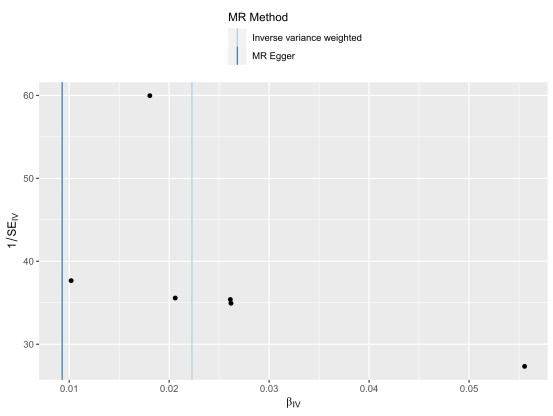


C D


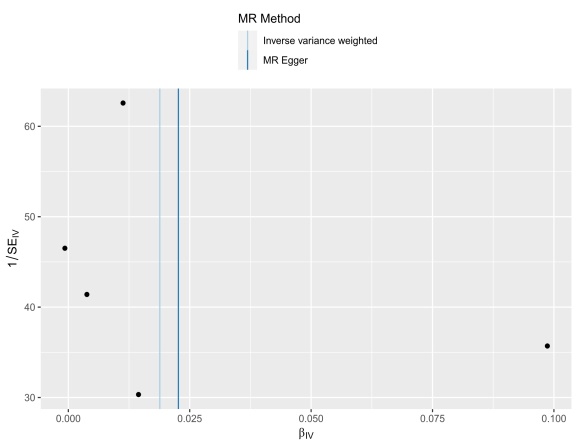

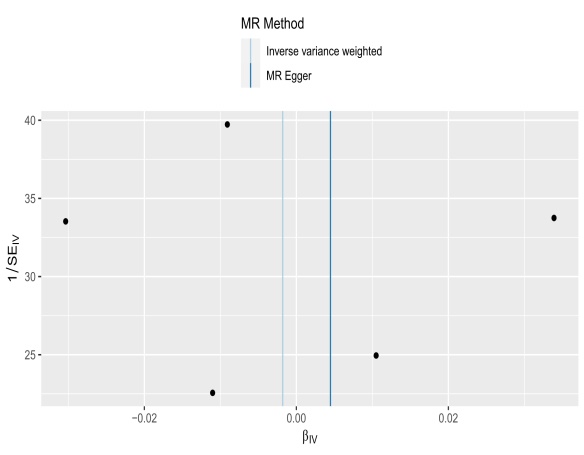


E F


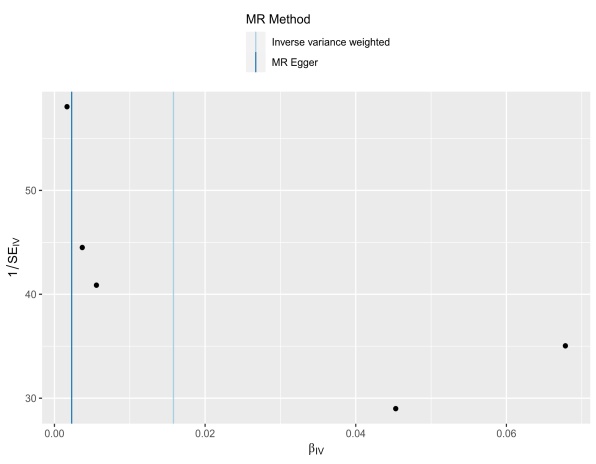

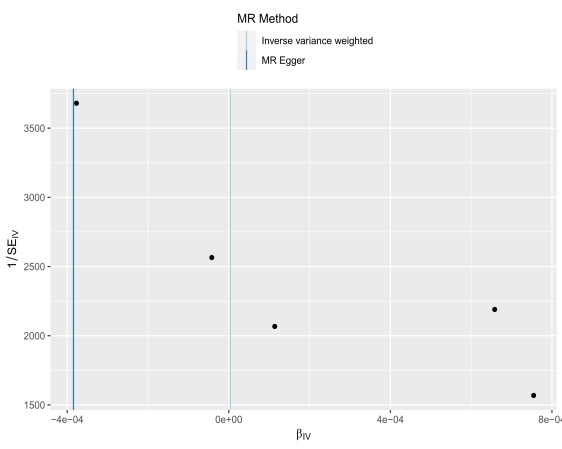


Supplementary Figure 2. Funnel plot for IVW and MR-Egger method. (A) AS and hypertension; (B) AS and heart failure. (C) AS and atrial fibrillation; (D) AS and coronary heart disease; (E) AS and myocardial infarction; (F) AS and peripheral artery disease.

A B


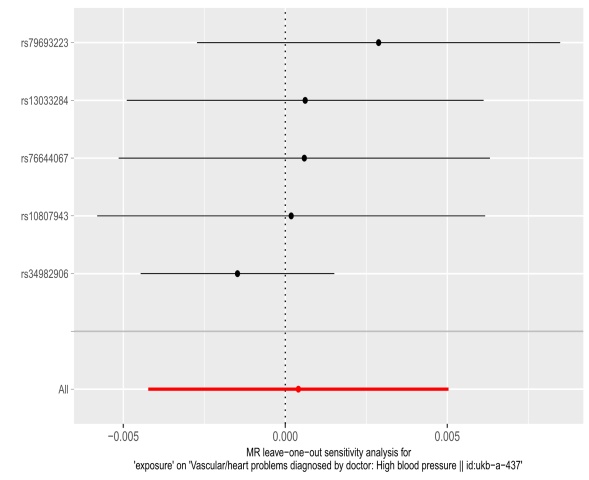

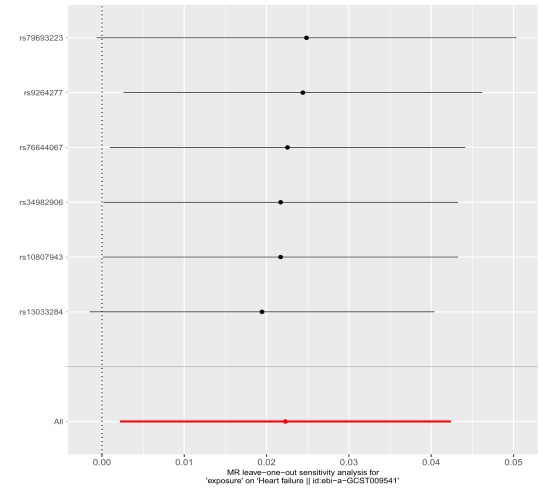


C D


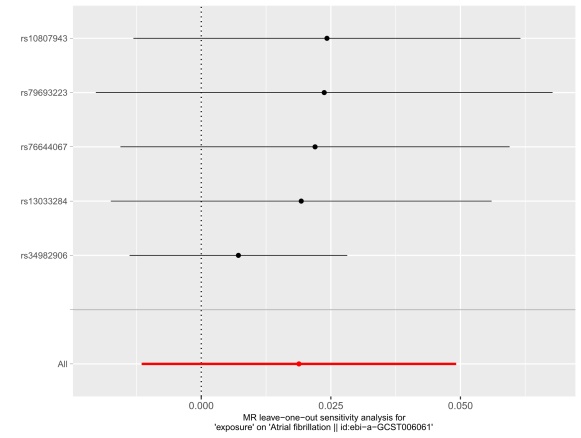

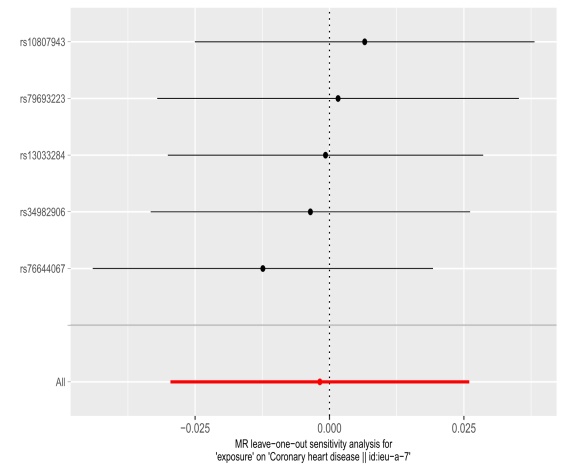


E F


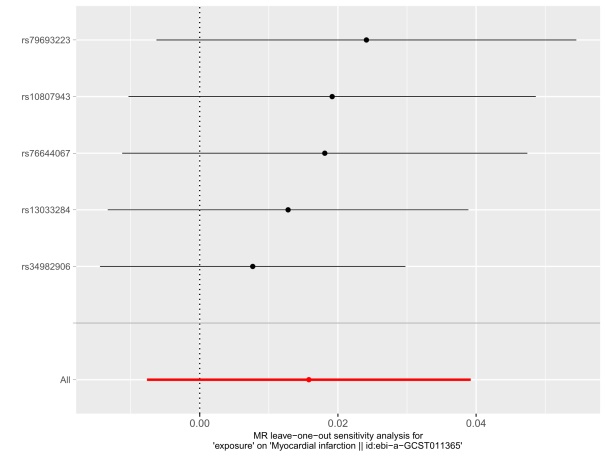

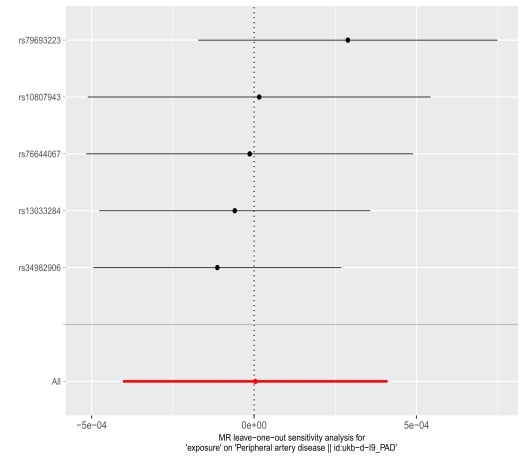


Supplementary Figure 3. Leave-one-out sensitivity analysis. (A) AS and hypertension; (B) AS and heart failure. (C) AS and atrial fibrillation; (D) AS and coronary heart disease; (E) AS and myocardial infarction; (F) AS and peripheral artery disease.
